# Am I Paid Well Enough to Be Diagnosed with COVID-19? Determinants of Gender Differences in Infection Detection Rate among Polish Working Age Population

**DOI:** 10.3390/jpm12050793

**Published:** 2022-05-14

**Authors:** Marcin Piotr Walkowiak, Dariusz Walkowiak

**Affiliations:** 1 Department of Preventive Medicine, Poznan University of Medical Sciences, 61-701 Poznań, Poland; 2 Department of Organization and Management in Health Care, Poznan University of Medical Sciences, 61-701 Poznań, Poland; dariuszwalkowiak@ump.edu.pl

**Keywords:** COVID-19, severe acute respiratory syndrome coronavirus 2 (SARS-CoV-2), COVID-19 testing, infection, COVID-19 diagnosis, public health

## Abstract

In comparison to Western European countries, Poland had a relatively lower percentage of its population diagnosed with COVID-19. Moreover, even the detected cases were not showing any pattern consistent with the expected chance of infection and were at best only remotely related to the severity of the illness that is known to increase with age. Instead, the crucial factor in detecting illness was whether the individual was likely to receive adequate compensation for being confined to their home, with employed women being the most likely to be diagnosed. In every Polish sub-region (powiat), in the 25–54 age group, the share of men diagnosed with COVID-19 was lower than that of women, with the missing share ranging from 8% to 36%. Based on the regression model (adjusted R² = 43.9%), there were relevant non-economic factors such as education, vaccination rate and increasing median age that were reducing this gap. However, the key factors, such as the share of population entitled to sick leave derived from employment rate, or the share of the self-employed population who were unlikely to receive adequate compensation, were related to economic incentives. It would seem that gender differences, in reaction to economic stimuli, widened the discrepancies, as the same factors were affecting women as well. While the testing rates in Poland, the lowest of all the EU countries, clearly played a role in creating the environment in which testing was perceived by the general population as somewhat optional, Polish citizens themselves through their actions aggravated the problem further, creating the impression of people receiving inadequate or no compensation for their time of self-isolation. In spite of well-intentioned government efforts to extend compensation to at least some groups, a significant share of the population clearly behaved as if they feared self-isolation more than the actual virus. Therefore, for both compliance and fairness purposes, both the severity of restrictions and the availability of compensation should be reconsidered.

## 1. Introduction

The World Health Organization (WHO) recognized diagnostic testing for SARS-CoV-2 as a critical component of the overall prevention and control strategy for COVID-19 [1]. There is no doubt that effective testing helps contain the spread of the pandemic and that such solutions should be implemented in all countries where possible and as soon as possible. Its aim was to protect the most vulnerable groups, but often also to take the strain off the local health systems. The latter issue, of course, indirectly influenced the possibility of helping not only people suffering from COVID-19, but also other patients.

The spread of the COVID-19 pandemic has resulted in unprecedented efforts to suppress consecutive waves but also to keep the healthcare system working, also in view of the need to ensure continuity of care for other diseases. These efforts differed in particular countries, but they were usually associated with various restrictions for citizens, including severe travel limitations and home confinement, or significantly restricted the out-of-home activities of residents. It should be remembered that the risks resulting from falling ill with COVID-19 varied in different age groups, but older people potentially faced the most serious consequences [2]. A significant part of the younger population could therefore consider the adopted restrictions too far-reaching. These efforts disrupted business operations, leading to enormous socio-economic costs and resulting in a devastating impact of the crisis [3,4,5]. Individual countries have started to implement various types of support systems for non-functioning branches of the economy. In turn, economic pressure from businesses, but also from citizens, prompted many governments, and later also corporations, to lift the restrictions. Safety strategies often included a 14-day quarantine, as recommended by the WHO, stating that “shortening the quarantine period will result in a larger proportion of contacts becoming infectious after leaving quarantine, but conversely may lead to greater compliance and result in a reduction of transmission” [6]. These quarantines in different countries were usually combined with entry and exit tests, where a positive test result prompted isolation until recovery. Only recently was it shown that “response system based on enhanced testing and contact tracing can play a major role in relaxing social distancing interventions” [7] and appropriately timed testing can make shorter quarantines effective [8].

However, it seems that this policy was implemented in many different ways in various countries. This was probably due to diverse local factors, and in some countries financial and organizational possibilities were also important. However, if we look at the strategy in this area in European Union (EU) countries only, then when analyzing only the frequency of testing, we will notice fundamental differences [9].

The testing policy cannot be discussed without referring to local conditions and, of course, to the number of infections in a given period of time. However, it is worth remembering that depending on the testing strategy adopted, the number of infections revealed will in many cases depend on the number of tests performed. In many cases, the observed differences in the percentages of positives tell us more about the local testing policy than about the actual number of infections. Differences in the number of SARS-CoV-2 infections may be attributed to racial, ethnic or socioeconomic disparities [10,11,12]. Globally, the gender differences in COVID-19 infection rates can be explained by women’s lower access to healthcare due to social norms and financial and non-financial barriers which may affect testing [13]. Interestingly, such differences were observed not only in poor countries but also in certain wealthy ones, such as the USA [12]. On the other hand, a study conducted in the Netherlands found no sex differences in COVID-19 testing in the general population [14].

However, despite the fact that as of December 2021, only 64.4% of adult Poles were vaccinated [15], the study commissioned by the Polish Ministry of Health [16] shows that at exactly the same time, 78.1% of Polish residents aged 20 or older had anti-SARS-CoV-2 antibodies (with a further 3.4% having reached a borderline score). Of those who did not get vaccinated, 53% had antibodies (positive or borderline).

The aim of the present study is to analyse gender differences in COVID-19 detection rates in the Polish population. Subsequently, economic incentive mechanisms will be analysed, and we will model the COVID-19 detection rate among Polish men and women in working age (25–54) based on socio-economic factors in the period 1 January 2021 to 21 March 2022. Finally, a linear regression model will be tested in spatial regression to filter out factors that could be potentially explained as unaccounted regional differences.

## 2. Methods

In order to avoid comparing informal local differences in testing policies, testing accessibility and genuine local infection rate differences, the study concentrates on gender differences in COVID-19 detection. Preliminary analysis and data visualization were created based solely on official infection statistics and relevant demographic data. The key explanatory analysis was conducted using backwards stepwise linear regression, initially simultaneously trying all the variables mentioned underneath, and subsequently testing for collinearity. Such a narrowed-down set of explanatory variables was tested one more time in a spatial regression model, and further backwards stepwise linear regression was applied to eliminate variables that have lost statistical significance after adjusting for spatial relations. Both models are presented.

A list of all registered COVID-19 infections in Poland from 1 January 2021 to 21 March 2022 was taken from the Ministry of Health of the Republic of Poland [15]. Each record contained information on gender, age, sub-region (powiat) and vaccination status. Of the total 4,716,312 records, there were 4,669,394 complete records of COVID-19 infections pertaining to the age group 25–54. The selection of the time period for analysis was based on data availability and the intent to concentrate on the time when the initial panic had already subsided and was slowly being replaced by pandemic fatigue, with people trying to continue normal life, despite the still-present forced isolation and quarantine restrictions. The age range was intentionally narrowed down to those old enough to no longer be in education, yet young enough not to fall in the high-risk category.

Data on the employment ratio, household size and the share of the population with higher education, sorted by sub-regions, were taken from the 2011 General Census [17]. Because of subsequent administrative reorganisation, we reduced the number of available sub-regions from 380 to 378. The number of visits to a general practitioner (GP) in the last available year 2020 and data on the percentage change in number of visits between the years 2019 and 2020, which could have been a marker of intentionally avoiding being diagnosed, were taken from Statistics Poland [17]. Data on the number of people who earn their living solely from their own farmland were taken from the Agricultural Census of 2020 and were converted to ratio per 1000 inhabitants. Data on the number of entrepreneurs were taken from the monthly report of Statistics Poland as of December 2020 on the number of registered businesses, excluding people solely farming [17]. These data were also converted to ratio per 1000 inhabitants.

Median age of sub-region population and gender ratio in the analysed group required to adjust the detection rate to the actual population composition were taken from Statistics Poland, as of end of 2020. The number of books borrowed from public libraries in that period per 1000 inhabitants was also used as a proxy of a more intellectual mindset of the local population.

Information on voter turnout from the second round of the presidential election of 2020, as a proxy of a civic mindset, was taken from the National Electoral Commission website [18]. Vaccination rate data, aggregated into age groups, were taken from the Ministry of Health website as of 30 June 2021. Of these, we chose for our model the age groups 20–39 and 40–59.

Variables in regression models, except for the constant ones, were required to be statistically significant, with *p* < 0.05, in order to be included and be subject to further analysis. Initial processing of raw data was conducted with Python (Pandas), statistical analysis was performed in Gretl 2019d, while spatial regression was calculated in GeoDa 1.18.0, which was also used for map generation.

## 3. Results

### 3.1. Cost Benefit Analysis of Being Diagnosed with COVID-19

There was a serious incurred cost of forced isolation, well exceeding symptomatic disease and likely to put perfectly healthy household members on weeks-long quarantines. Violating those restrictions was subject to very severe fines, which—if challenged in court—were often even found to be excessive. By contrast, not seeking a diagnosis was perfectly legal, which was creating a perverse incentive structure.

In Poland, there were two main types of self-isolation with slightly different legal consequences. There was isolation (*izolacja*) for people who tested positive for COVID-19, and quarantine (*kwarantanna*) for those who either had had close contact with an infected person, had crossed the border, or had requested a test but did not receive its results. Under general rules [19], at the beginning of the analysed period, isolation lasted for at least 13 days for symptomatic cases, with the requirement of the last 3 days without any symptoms, and for 10 days for asymptomatic cases. On May 6 2021, the isolation for symptomatic cases was shortened to 10 days, on the condition that the last day is without fever. Regardless of the length of isolation, it was counted from the day of receiving the test results, which was typically day after sample collection. This fact could lengthen patient isolation purely because of the lack of laboratory processing capacity. On 15 February 2022, isolation was shortened to 7 days, counted from the day of actually taking the test.

For almost the whole analysed period there was a 10-day quarantine in the case of detected contact with an infected person (shortened to 7 days on 25 January 2022), and 7 days from the end of isolation of an infected household member. On 11 February 2022, quarantines based solely on contact were abolished. On 15 February 2022, quarantines were shortened to the period of isolation of sick household members. People who were either vaccinated or tested positive for COVID-19 within last 6 months were exempted from quarantine. Additionally, from 15 December 2021, such people were technically no longer exempt from quarantine in the case of having an infected household member, though in practice were released from it as soon as they receive a negative test result. There was no special quarantine exemption for children who were too young to be vaccinated.

There were two other notable unintended incentives within the system. While the government was officially encouraging people to take a test on the 7th day of a 10-day quarantine, that would not end it, so it was exactly the least advantageous option. If one wanted to get tested, then regardless of having any symptoms it was advantageous to immediately request a test, as isolation would not be much longer, while one would at least receive a COVID-19 infection certificate. However, if one was sent a test by their GP, and its positive result would mean numerous household members had to quarantine, they was incentivised not to do it, or at least postpone until the test would turn out to be negative. The pending test would keep him/her on quarantine, but at least his/her household members would remain free.

For high-risk people, the need to receive medical care was a crucial advantage, overruling other factors. In the case of people in employment or of students in universities that were strict about medical leave documents, they needed to meet with their doctor. However, even such people had some leeway, whose creation was, paradoxically, caused by the wish to combat the spread of COVID-19—they could have requested a phone consultation with their doctor and downplay their symptoms towards a common cold. While theoretically medical professionals were required by law to send the patients suspected of having COVID-19 to be tested, the regulation was relatively vague, and, based on the low requested number of tests and at the same time the high positive rate of such tests, it was predominately interpreted rather narrowly.

The government tried to make COVID-19 isolation and quarantine restrictions more palatable to the citizens by extending sick leave, though the created system was patchy. People on quarantine, isolation or childcare leaves were paid 80% of their salary (which is usual for sick leave in Poland), people suffering from COVID-19 who were fighting with pandemic and were infected on their job were paid 100%.. Both quarantine and isolation were effectively treated like any other sick leave, so in a year first 33 days (or 14 days in case of employees being at least 50 years old) were financed by the employer, and only subsequent part was financed by the government. In contrast with pre-pandemic rules, childcare leave was extended and fully financed by government, but it was still paid at 80%.

Entrepreneurs were even less fortunate. Firstly, they had legal and contractual obligations that remained effectively unchanged regardless of illness. Secondly, although they were allowed to pay very low preferential social security insurance during the first 2.5 years of their business, their entitlements were nevertheless proportional to their contribution. After that period, their social security contribution and allowances in general were such as if they were earning the equivalent of 60% of the average salary in Poland, though the payment of contribution entitling them to sick leave remained elective and relatively unpopular. Freelancers and people earning from minor civil law contracts were either exempt or allowed not to pay sick leave contribution; thus, they were unlikely to receive any compensation.

The insurance scheme for farmers before the pandemic was characterised by very low contributions and allowances, as in order to receive payment one was required to be sick for at least 30 days and even then, they received merely PLN 15 (less than EUR 4) per day. The government noticed that such a scheme was unlikely to motivate people to seek diagnosis, so for COVID-19 patients, the requirement of minimum illness length was lifted, and farmers sent to quarantine, regardless of its length, were paid the sum equivalent to half minimum salary and exempted from income tax.

### 3.2. COVID-19 Infection Detection Rate Based on Age and Gender

**Figure 1:**
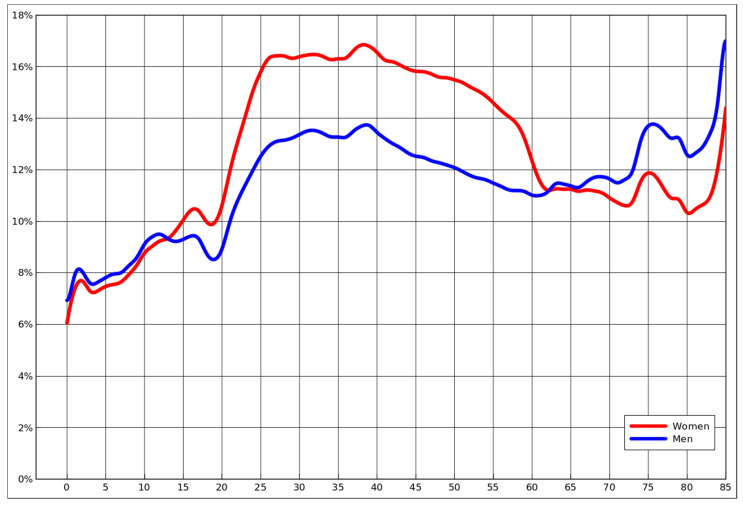
The percentage of each yearly cohort that was diagnosed with COVID-19, by gender, for the period 1 January 2021 to 21 March 2022. Due to data availability, values are smothered with 3 year moving average. Age brackets of 85 and above are grouped under 85.

From a purely epidemiological standpoint, one would expect in general that the most infected group would be those unvaccinated, not wearing masks and less careful because of low risk of a severe illness, with males and females equally likely to become infected [20]. However, as presented in Figure 1, based on actual detection rate, that is most certainly not the case. Young children, even though those below age 5 that were not even required to wear masks, while vaccination of those aged 5–11 started near the end of the analysed period (14 December 2021), were exactly the group with the lowest percentage of diagnosed cases.

While the detection rates among boys and girls were almost identical, only slightly higher for boys, they changed and diverged in teenage years. The rate of infection starts to skyrocket exactly at the age when people enter the job market, roughly stabilises on clearly diverging levels at the moment when people typically end their tertiary education, and then slowly declines for middle-aged people. The gender difference suddenly drops near the age of 60, which is the Polish retirement age for women. Subsequently it actually becomes slightly higher for men, who, based on prior mortality studies, were found to be generally more vulnerable [2].

**Figure 2:**
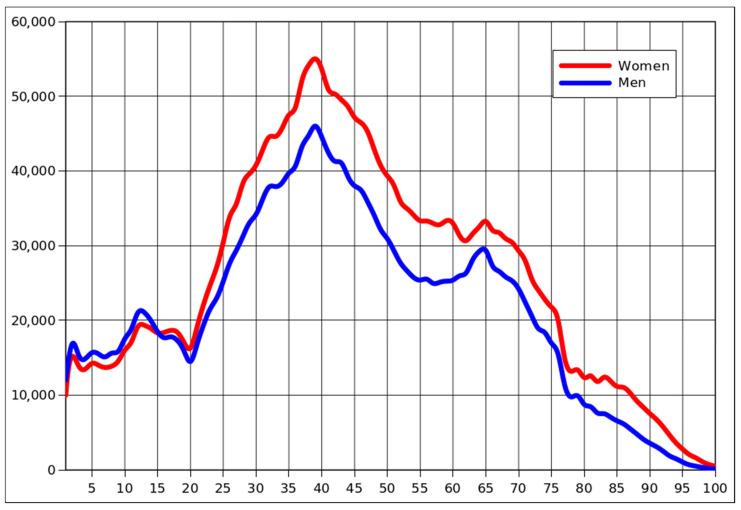
The number of cases in each yearly cohort that was diagnosed with COVID-19, by gender, for the period 1 January 2021 to 21 March 2022.

A similar pattern is emerging in Figure 2, presenting the nominal number of detected cases in all age groups. Except for the obvious distortion caused by the baby boom and its effects, there was also the issue of men dying earlier, which led to the situation where women predominated since adolescence, without exception, among those diagnosed with COVID-19.

**Figure 3:**
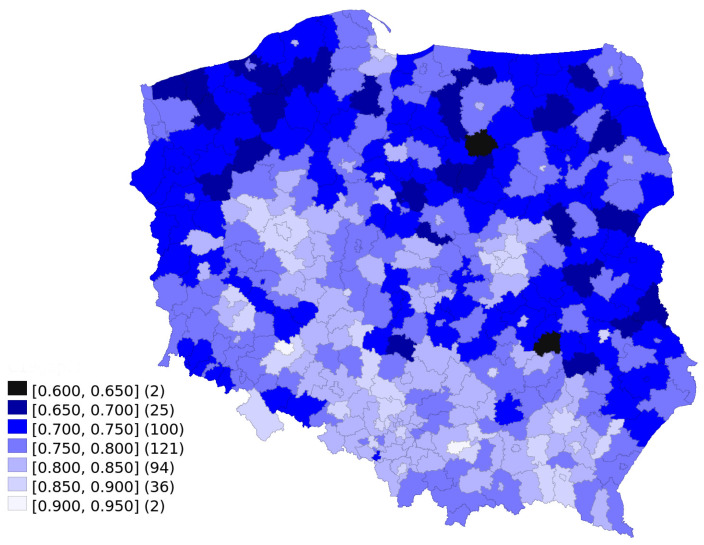
Male to female ratio of COVID-19 detection among the working-age (25–54 years) population of Poland for the period 1 January 2021 to 21 March 2022, corrected for gender imbalance.

Figure 3 presents sex differences in COVID-19 detection among the working age population of Poland, divided into sub-regions. The observed gender differences in COVID-19 detection were characterised by some regional variability, with a more balanced detection rate in central and southern Poland. Moreover, the gap in detection rates between men and women was clearly lower in cities, towns and suburbs than in the country. In all sub-regions, the number of men diagnosed with COVID-19 was lower than could be expected if the detection rate was identical for both genders. The missing detection share varies between 8% and 36%.

### 3.3. Modelling Gender Differences in COVID-19 Detection Rate

In Table 1, we present models explaining the gender gap in COVID-19 detection. As in Figure 3, “0” would mean than no men were diagnosed with COVID-19, while “1” would mean exactly the same ratio for both genders.

In the model without spatial lag, all variables except for constant are significant with *p* < 0.001. Gender gap in the detection of COVID-19 is reduced by the vaccination rate and median age. The share of people with higher education, as well as the employment ratio and household size (with all these data derived from the 2011 census), narrow this gap. On the other hand, being self-employed, regardless of whether in agriculture or not, contributes to widening the gap. The same pattern repeats itself for the model with spatial lag, except in that model, median age and household size lose their statistical significance. Such variables as the number of visits to a GP, the change in frequency of these visits, the number of books borrowed per 1000, or civic participation (measured through voter turn-out) were not statistically significant.

## 4. Discussion

Poland had the lowest number of officially recorded tests taken among all the EU countries: as of the moment of effectively ending easily available testing, it was less than a million tests per million inhabitants [21]. Additionally, the initial limited enthusiasm of the population to vaccinate, as well as the lack of political will to compel the population to vaccinate [22,23,24] and the low effectiveness of later efforts of booster vaccination rate, all jointly created an environment in which there was a significant share of unvaccinated people [25,26,27], who—while wishing to avoid a PCR test—did not mind being later tested for antibodies. While such a situation evidenced the failure of a policy intended to contain the spread of the virus, from our research perspective this was a perfect opportunity for a natural experiment on individual motivation, since testing for COVID-19 became more of a personal choice.

The government correctly identified the existence of a problem and tried to mitigate it by slightly raising the number of (paid) sick days for coronavirus reasons. Based on our research, the policy was a small step in the right direction, as there was a higher detection rate among those compensated. The very idea that people behave in their self-interest is not new and is a base assumption of economic models. However, we made a new finding that instead of the generally mentioned issues, such as the actual spread of the disease or the availability of testing, economic self-interest could have become the predominant factor in the diagnosis of infectious disease, and this is a key finding for future policies.

An issue that needs to be further taken into account is the observed difference in the immune responses between men and women [28,29,30]. While there exist stable immunological differences between the sexes that do not change over the entire lifespan, there are also others, manifested only in the time period after puberty but before reproductive senescence. The latter age-related differences imply an interplay of genetic and hormonal factors. All these differences, however, regardless of the patients’ age, have a bearing on the clinical aspects of the antiviral reaction and tissue resilience in COVID-19 patients. Not to be overlooked is also the finding that older men appear especially prone to severe COVID-19 disease, with higher mortality reported in that group [31,32]. Moreover, our previous study of excess mortality in Poland found significant differences between the sexes also in younger age groups [2]. It should be considered whether organizing the testing system in such a way that encouraged the patients to take into account, apart from strictly medical factors, also economic ones, could not lead to negative results, including an increase in mortality caused by the failure to detect the virus.

The mere fact that various factors contribute to the emergence of differences between social groups, also between the sexes, is not surprising [33,34,35]. However, the only two statistically significant variables in our study that seem to be indeed sociological in nature were higher education and vaccination rate in the 20–39 age group. Among university-educated people, the gender gap was much smaller, and this factor had high impact. Much weaker was the impact of vaccination, and this seemed related to the local perception of the seriousness of the problem. The median age was most probably increasing average illness severity, forcing men to seek medical help and thus reducing gender imbalance among working age population.

All the remaining variables do appear to be linked with individual interests, based on the incentive structure created. The bigger the household, the lower gender imbalance—either no-one was tested, in order to avoid problems, or everyone was. The higher the employment rate, the lower the gender gap, as there was a higher share of people entitled to sick allowance. As farmers and businesspeople were receiving only meagre sick compensation, they had no incentive to be diagnosed; the impact of each individual farm or registered business on gender imbalance in a sub-region is actually quite strong.

Still, before the pandemic, in the years 2015–2019, the men-to-women ratio of work absenteeism caused by sickness among those aged 20-64 was 65.2% [36]. On the other hand, the men-to-women ratio of detection rate of COVID-19 in the analysed 25–54 age group was 81.6% (the ratio was the same for the 25–64 age group). Neither of those values are adjusted for size of working age population, so in both cases the actual ratio would be skewed even more towards women seeking medical leave in case of a less severe illness. One could have seen it as a kind of improvement in relation to the detection of other illnesses, though there is a big caveat. If testing for COVID-19 in the case of a minor infection was effectively optional, and women were more likely to see a doctor with a minor illness, then such a less skewed ratio would be the natural outcome.

Our model only calculates the gender gap, and not the actual number of missing cases. Women were just less sensitive than men to those motivating factors, so a huge gender gap in a sub-region most likely means that women there were actually seriously under-diagnosed in comparison with those in more gender-balanced regions. Working-age (25–54) men were still more likely to be diagnosed than young people (18–24) of both genders. This finding further reinforces the argument about the paramount importance of economic incentives, as cohorts composed mostly of students were even less likely to be diagnosed.

In this particular case some minor adjustment of policy would not have changed situation diametrically. The problem was that only a small share of the population had their inconvenience compensated enough to seek being tested, and those people tended to be employed women whose households were apparently not endangered by quarantine.

The simplest answer would be to pay better, possibly by giving everyone some minimum COVID-19 sick allowance, regardless of their age and employment status, or by using a more sophisticated instrument based on the socio-economic status of the person who is isolated or quarantined. Could the government offer such money that the overwhelming majority of population would happily book a PCR test whenever they feel a minor pain in the throat? In the case of the well-contained pandemic, this would be theoretically doable, though it would also lead to a different set of perverse incentives. However, if one objects to that on the grounds that the cost would be horrendous, then one inadvertently admits that the cost already incurred was horrendous, only most of it was borne by those isolating and quarantined.

From the cost–benefit perspective, if no increase in benefits was possible, then one could have at least tried to reduce the costs to individual. Isolation and quarantines were initially implicitly based on pessimistic estimates of the possible time of an individual being infectious. While this might look as erring on the side of caution, which seemed justifiable, the hidden cost was discouraging highly infectious people from testing. COVID-infected people would look at the time of isolation differently if it were merely a day or two longer than the time they would stay bedridden anyway. Making the restrictions more palatable, flexible and compatible with their pre-pandemic daily routine would encourage them to be diagnosed, which was clearly the cheapest option.

The fourth avenue for improving detection rates was by changing testing policy. Under existing rules, in most cases the quarantine was a blunt instrument. Except for a few cases, such as border crossing or being a vaccinated household member of an infected person, a negative test result did not release an individual from quarantine. A better-designed system would compel people to get tested. Instead of unconditional quarantine, the system could have been sending people to test under threat of quarantine, which would be extended onto the rest of their household if no test was taken within a prescribed time. The official policy would not have to rely on documented contacts; instead, it could have been targeting people living in a virus hotspot, or even selecting them at random, to gauge the actual virus prevalence and by chance detecting positive cases.

## Figures and Tables

**Table 1:** Male-to-female ratio of COVID-19 detection among the working-age (25–54 years) population in in the age bracket 25–54 in the period 1 January 2021 to 21 March 2022, corrected for gender imbalance.

Variable	Model without Lag	Model with Spatial Lag
Const	−0.201 (0.114)	0.212 (0.0392) ***
Spatial lag		0.620 (0.0501) ***
Vaccinated among 20–39	0.136 (0.0445) ***	0.0792 (0.0382) *
Higher education	0.529 (0.0813) ***	0.368 (0.0567) ***
Employment rate	0.517 (0.0846) ***	0.0993 (0.0420) *
Median age	0.0113 (0.00190) ***	
Household size	0.0835 (0.0133) ***	
Number of businesses per 1000 Number of farmers per 1000	−0.826 (0.173) *** −0.698 (0.0896) ***	−0.302 (0.152) * −0.213 (0.0724) **
Adjusted R²	43.9%	53.3%

* *p* < 0.05, ** *p* < 0.01, *** *p* < 0.001, standard deviation in brackets.

## Data Availability

https://bdl.stat.gov.pl/BDL/dane/podgrup/tematbasiw.mz.gov.pl/index.html#/visualization?id=3761pkw.gov.pl (accessed on 7 May 2022).

## Abbreviations

COVID-19: coronavirus disease 2019; European Union: EU; GP: general practitioner; WHO: World Health Organization.

## References

[1] WHO Recommendations for National SARS-CoV-2 Testing Strategies and Diagnostic Capacities. https://apps.who.int/iris/rest/bitstreams/1352897/retrieve.

[2] Walkowiak M.P., Walkowiak D. (2022). Underestimation in Reporting Excess COVID-19 Death Data in Poland during the First Three Pandemic Waves. Int. J. Environ. Res. Public Health.

[3] Nicola M., Alsafi Z., Sohrabi C., Kerwan A., Al-Jabir A., Iosifidis C., Agha M., Agha R. (2020). The Socio-Economic Implications of the Coronavirus Pandemic (COVID-19): A Review. Int. J. Surg..

[4] Martin A., Markhvida M., Hallegatte S., Walsh B. (2020). Socio-Economic Impacts of COVID-19 on Household Consumption and Poverty. Econ. Disaster. Clim. Chang..

[5] Šteinbuka I., Austers A., Barānovs O., Malnačs N. (2022). COVID-19 Lessons and Post-Pandemic Recovery: A Case of Latvia. Front. Public Health.

[6] Considerations for Quarantine of Contacts of COVID-19 Cases. https://www.who.int/publications-detail-redirect/WHO-2019-nCoV-IHR-Quarantine-2021.1.

[7] Aleta A., Martín-Corral D., Piontti A.P.Y., Ajelli M., Litvinova M., Chinazzi M., Dean N.E., Halloran M.E., Longini I.M., Merler S. (2020). Modeling the Impact of Testing, Contact Tracing and Household Quarantine on Second Waves of COVID-19. Nat. Hum. Behav..

[8] Wells C.R., Townsend J.P., Pandey A., Moghadas S.M., Krieger G., Singer B., McDonald R.H., Fitzpatrick M.C., Galvani A.P. (2021). Optimal COVID-19 Quarantine and Testing Strategies. Nat. Commun..

[9] Ritchie H., Mathieu E., Rodés-Guirao L., Appel C., Giattino C., Ortiz-Ospina E., Hasell J., Macdonald B., Beltekian D., Roser M. (2020)—“Coronavirus Pandemic (COVID-19)”. Published online at OurWorldInData.org. https://ourworldindata.org/coronavirus.

[10] Gender and Health. https://www.who.int/health-topics/gender.

[11] Muurlink O.T., Taylor-Robinson A.W. (2020). COVID-19: Cultural Predictors of Gender Differences in Global Prevalence Patterns. Front. Public Health.

[12] Mannheim J., Konda S., Logan L.K. (2022). Racial, Ethnic and Socioeconomic Disparities in SARS-CoV-2 Infection amongst Children. Paediatr. Perinat. Epidemiol..

[13] Aleksanyan Y., Weinman J.P. (2022). Women, Men and COVID-19. Soc. Sci. Med..

[14] Ballering A.V., Oertelt-Prigione S., olde Hartman T.C., Rosmalen J.G.M. (2021). Sex and Gender-Related Differences in COVID-19 Diagnoses and SARS-CoV-2 Testing Practices During the First Wave of the Pandemic: The Dutch Lifelines COVID-19 Cohort Study. J. Womens Health.

[15] BASiW-Statystyki Zakażeń i Zgonów z Uwzględnieniem Szczepienia Przeciw COVID-19. https://basiw.mz.gov.pl/index.html#/visualization?id=3761.

[16] Narodowy Instytut Zdrowia Publicznego Ogólnopolskie Badanie Seroepidemiologiczne COVID-19 OBSER-CO Podsumowanie wyników III tury badania. https://www.pzh.gov.pl/download/29904/.

[17] GUS-Bank Danych Lokalnych. https://bdl.stat.gov.pl/BDL/dane/podgrup/temat.

[18] Państwowa Komisja Wyborcza. https://pkw.gov.pl.

[19] Rozporządzenie Ministra Zdrowia z Dnia 25 Lutego 2021 r. w Sprawie Chorób Zakaźnych Powodujących Powstanie Obowiązku Hospitalizacji, Izolacji Lub Izolacji w Warunkach Domowych Oraz Obowiązku Kwarantanny Lub Nadzoru Epidemiologicznego. https://eli.gov.pl/eli/DU/2021/351/ogl.

[20] Arnold C.G., Libby A., Vest A., Hopkinson A., Monte A.A. (2022). Immune Mechanisms Associated with Sex-Based Differences in Severe COVID-19 Clinical Outcomes. Biol. Sex. Differ..

[21] Data on Testing for COVID-19 by Week and Country. https://www.ecdc.europa.eu/en/publications-data/covid-19-testing.

[22] Walkowiak M.P., Walkowiak D. (2021). Predictors of COVID-19 Vaccination Campaign Success: Lessons Learnt from the Pandemic So Far. A Case Study from Poland. Vaccines.

[23] Walkowiak M.P., Walkowiak J.B., Walkowiak D. (2021). COVID-19 Passport as a Factor Determining the Success of National Vaccination Campaigns: Does It Work? The Case of Lithuania vs. Poland. Vaccines.

[24] Grabowski J., Witkowska N., Bidzan L. (2021). Letter to the Editor: Excess All-Cause Mortality during Second Wave of COVID-19—The Polish Perspective. Eurosurveillance.

[25] Jarynowski A., Wójta-Kempa M., Płatek D., Czopek K. (2020). Attempt to Understand Public-Health Relevant Social Dimensions of Covid-19 Outbreak in Poland. SSRN.

[26] Sowa P., Kiszkiel Ł., Laskowski P.P., Alimowski M., Szczerbiński Ł., Paniczko M., Moniuszko-Malinowska A., Kamiński K. (2021). COVID-19 Vaccine Hesitancy in Poland-Multifactorial Impact Trajectories. Vaccines.

[27] Walkowiak M.P., Domaradzki J., Walkowiak D. (2022). Better Late Than Never: Predictors of Delayed COVID-19 Vaccine Uptake in Poland. Vaccines.

[28] Bunders M.J., Altfeld M. (2020). Implications of Sex Differences in Immunity for SARS-CoV-2 Pathogenesis and Design of Therapeutic Interventions. Immunity.

[29] Takahashi T., Iwasaki A. (2021). Sex Differences in Immune Responses. Science.

[30] Bechmann N., Barthel A., Schedl A., Herzig S., Varga Z., Gebhard C., Mayr M., Hantel C., Beuschlein F., Wolfrum C. (2022). Sexual Dimorphism in COVID-19: Potential Clinical and Public Health Implications. Lancet Diabetes Endocrinol..

[31] Klein S.L., Flanagan K.L. (2016). Sex Differences in Immune Responses. Nat. Rev. Immunol..

[32] Scully E.P., Haverfield J., Ursin R.L., Tannenbaum C., Klein S.L. (2020). Considering How Biological Sex Impacts Immune Responses and COVID-19 Outcomes. Nat. Rev. Immunol..

[33] Laaksonen M., Martikainen P., Rahkonen O., Lahelma E. (2008). Explanations for Gender Differences in Sickness Absence: Evidence from Middle-Aged Municipal Employees from Finland. Occup. Environ. Med..

[34] Xue Y., Kristiansen I.S., de Blasio B.F. (2010). Modeling the Cost of Influenza: The Impact of Missing Costs of Unreported Complications and Sick Leave. BMC Public Health.

[35] Santos J.R., May L., Haimar A.E. (2013). Risk-Based Input-Output Analysis of Influenza Epidemic Consequences on Interdependent Workforce Sectors. Risk Anal..

[36] Statistics|Eurostat. https://ec.europa.eu/eurostat/databrowser/view/lfsi_abs_q_h/default/table?lang=en.

